# Influence of operator’s professional experience in the postoperative course after surgical extrac-tion of the impacted lower third molar: A pilot study

**DOI:** 10.4317/jced.56549

**Published:** 2020-09-01

**Authors:** Juan-Antonio Ruiz-Roca, Benjamin Donoso-Martínez, Susana Ameneiros-Serantes, Yolanda Martínez-Beneyto, Diego Salmerón-Martínez, Cosme Gay-Escoda

**Affiliations:** 1 DDS, MDS, PhD. Asisstant Professor. Faculty of Medicine-Dentistry - University of Murcia (Spain). Researcher of University Institute for Research in Aging-University of Murcia, Spain; 2 DDS. Private practice. Murcia, Spain; 3 DDS, MS, PhD. Private practice. Narón, A Coruña, Spain; 4 DDS, MS, PhD. Associate Professor. Faculty of Medicine-Dentistry. Department of Dermatology, Estomatol-ogy and Radiology. University of Murcia, Spain; 5 PhD. Associate Professor, Department of Health and Social Sciences, University of Murcia (Spain); IMIB-Arrixaca, Murcia, Spain; CIBER Epidemiología y Salud Pública (CIBERESP), Spain; 6 MD, DDS, MS, PhD, EBOS, OMFS. Chairman and Professor of Oral and Maxillofacial Surgery, University of Barcelona, Director of the Master Degree Program in Oral Surgery and Implantology, EFHRE International University, Coordinator & Researcher of the “Institut d’Investigació Biomédica de Bellvitge” (IDIBELL Insti-tute), L’Hospitalet de Llobregat, and Head of Oral and Maxillofacial Surgery Department, Centro Médico Teknon, Barcelona, Spain

## Abstract

**Background:**

Third molars are present in 96.6% of humans, although they do not always erupt completely. Between 9.5% and 73% of them remain impacted. Surgical removal of impacted third molars is the most common practice in oral and maxillofacial surgery. This procedure results in traumatism and, consequently, the postoperative phase will involve symptomatology. It is uncommon to find studies that directly relate postoperative symptomatology and the operator’s experience. The aim of this study was to determine the differences regarding postoperative symp-tomatology in patients undergoing the bilateral extraction of lower impacted third molars and according to the operator’s experience.

**Material and Methods:**

A prospective cohort double-blind study was conducted in 50 healthy patients (100 molar extractions) to whom both lower third molars were removed by two dentists with different degree of professional experience. The extractions were randomly assigned with a split–mouth design. If an operator extracted the lower third molar on one side, the other operator extracted the contralateral one. The variables studied after four days of postoperative period were Pain (EVA scale), Inflammation and Trismus, in addition to intraoperative time and local anesthesia administered.

**Results:**

Statistically significant differences were detected in the time of intervention and in trismus, since the most experienced operator always needed less time and caused higher degree of trismus. However, this does not entail more inflammation or pain in patients, so there are no relevant differences between operators with more or less experience (*p*>0.05).

**Conclusions:**

The postoperative period is more favorable for the most experienced operator, although the results do not vary in a relevant manner between them.

** Key words:**Preemptive analgesia, dental extraction, cyclooxygenases, real-time polymerase chain reaction.

## Introduction

The extraction of the third molar (M3) is found among the most frequent procedures performed in Oral and Maxillofacial Surgery. In the United Kingdom one million extractions are performed annually and about 5 mil-lion in the U.S. (1). The upper M3s are impacted in a greater proportion with a prevalence in young adults that reaches up to 65-73%, according to the consulted literature (2) and with no gender differences (3).

If M3 is asymptomatic and there is another pathology such as caries or a lesion presumptively diagnosed as a cyst, etc., the American Association of Oral and Maxillofacial Surgeons (AAOMS) recommends preventative removal or, if not removed, clinical and radiological follow-up by a specialist on an annual basis (4). Other au-thors, however, advocate a waiting attitude until clinical signs and symptoms lead to the need for extraction. Numerous studies such as those by Alves-Pereira *et al.* (5), state that between 18 and 40% of asymptomatic M3s are extracted. As a matter of fact, for Fuster-Torres *et al.* (6) preventative extraction is the most common indica-tion, followed by orthodontic reasons, pericoronitis and caries.

The extraction of the impacted M3 is associated with a series of immediate postoperative reactions such as pain, inflammation and trismus, and possible long-term events due to both the operative difficulty caused by the impaction of the molar and the anatomical characteristics of the operative field, affecting, thus, the patients’ quality of life (7).

The complication rate ranges between 2.6 and 30.9%, being the outcomes influenced by various factors such as the age and health status of patients, gender, race, weight, body mass index, the degree of impaction of the third molar, proximity to the inferior dental nerve canal, smoking habits, consumption of contraceptive drugs, level of oral hygiene, surgical technique and the operator’s experience inter alia (8). Thus, prior to addressing a surgical extraction the degree of difficulty should be assessed.

In order to assess the position of the M3 the classification proposed by Pell and Gregory (9) is traditionally used. Other indices based on its position in space, and with the help of radiographs—have been proposed for guidance concerning the difficulty to remove the M3, as the method described by Koerner *et al.* (10).

Although many demographic, clinical, operative and anatomical factors influence postoperative symptomatology (8-15), we are to focus on those referring to the operator’s degree of experience. A surgeon with greater experience than another’s, although with the same level of training, is expected to obtain more favorable results in terms of operative times and, therefore, the postoperative quality of life of patients is expected to be better. As mentioned above, there are other factors that also influence and consequently lead us to think that, in principle, the expertise of operators is not a determining factor for the severity of postoperative symptomatology.

Given the lack of studies evaluating the influence of the surgeon’s expertise when removing the impacted lower third molar, we intend to conduct a research relating the time used by operators to perform these procedures considering their expertise and the degree of difficulty of these surgeries, allowing determining, thus, the different postoperative course in terms of pain, inflammation and trismus between the groups of patients operated on by each surgeon.

Some studies (13) show that, if the M3 is extracted by specialists with extensive experience, there is a significant reduction in the time required for surgery. The difficulty and postoperative factors entail that the surgeon’s therapeutic decision must always be based on a sound scientific basis and that the technique to be performed must be as conservative and noninvasive as possible. For this reason, the surgeon’s experience influences the degree of difficulty of the extraction and planning, what helps reduce both the intraoperative time and possible postoperative complications.

The main objective of this study was to determine, depending on the operator’s degree of expertise, whether there were significant differences in the postoperative course of patients undergoing bilateral extraction of the impacted lower third molar. To this end, possible differences between the two operators were analyzed considering the total time elapsed in each surgery, course of postoperative inflammation, assessment of trismus and pain reported by patients at day 4 post-surgery.

## Material and Methods

A prospective cohort double-blind study was conducted from January to June 2018 on 50 healthy patients from a private clinic and whose both lower third molars were removed by two dentists with different number of years of professional experience. The most experienced surgeon was called ‘operator 1’ (Op1) and the least experienced ‘operator 2’ (Op2). Both professionals had completed the same master’s degree in Oral Surgery and Orofacial Implantology (University of Barcelona) and there were 10 years of difference between their work experiences.

The study protocol complies with the guidelines marked by the Declaration of Helsinki and was approved by the Clinical Research Ethics Committee of the University of Murcia (ID: 1909/2018).

Inclusion Criteria were: 1) patients aged between 18 and 40 years; 2) third molars positioned at an angle greater than 20 degrees with regard to the axial plane of the second adjacent molar; 3) mesioangular or horizontal position; 4) position B, C, class I and II of the classification by Pell and Gregory, which translates into a degree of complexity 1 (slightly difficult), 2 (moderately difficult) or 3 (very difficult) according to Koerner’s surgical difficulty index; 5) no pathological history of interest (ASA I or II, in accordance with the American Society of Anesthesiologists) nor medically compromised (16).

Exclusion Criteria were: 1) patients with systemic pathology (ASA higher than II), pregnant and allergic to anesthetic, antibiotic or anti-inflammatory drugs; 2) no more than three points of difficulty between the two third molars (according to Koerner’s surgical difficulty index); 3) patients who do not need to undergo any ostectomy or odontosection of crown; 4) patients being treated with NSAIDs (nonsteroidal anti-inflammatory drugs) or analgesic agents within 15 days prior to surgery, and finally, 5) failure to accept and sign informed consent form.

-Study Design

This is a pilot study and the patients were operated on randomly an online service (www.randomization.com) was used without the patient ever knowing the category or rank of the surgeon who was to perform the surgery. Each of the operators (1 and 2) performed the surgery and extracted one lower third molar one month apart (recovery time). The split–mouth design was utilized, i.e. each patient’s M3 (symmetrical and with the same complication) was operated on by a different surgeon. Both of them were right-handed and always performed the surgery from the right side of the patient, regardless of the M3 to be extracted.

-Operative Records

After the surgery all patients were given written instructions about the postoperative medication.

A third researcher (operator 3) gave each patient a leaflet to record the intensity of pain by using a visual analogue scale (VAS) (17), every hour for the first six hours after tooth removal, and in the morning and at night on a daily basis until day 4. The patients had to write down the number of analgesic capsules they took at all times.

**Figure 1:**
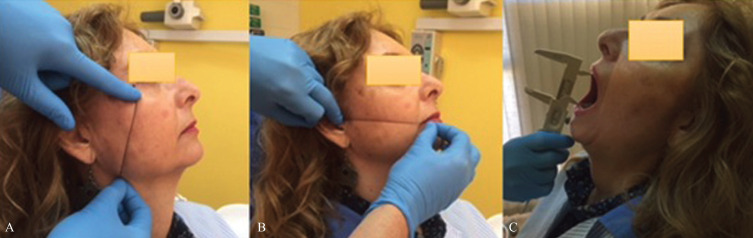
The distance from the external edge of the eye to the cutaneous area of the gonion (mandibular angle) (A) and the distance from the tragus to the oral commissure (B), as well as trismus (C).

Inflammation or edema was objectified by following the technique described by Amin and Laskin in 1983 (18), measuring by means of 2/0 dental floss of 20cm in length the distance from the external edge of the eye to the cutaneous area of the gonion (mandibular angle), and the distance from the tragus to the oral commissure previously marking the anatomical landmarks on the skin with a fine-tip permanent marker pen rather than hen-na (Fig. 1). These measurements were taken by researcher 3 just before and 72 hours after surgery. Depend-ing on the obtained results, these were classified according to the increase in measurement: no inflammation (0mm), mild inflammation (1–5mm), moderate inflammation (6–10mm) and severe inflammation (>10mm). Prior to beginning this phase of the study, training and calibration of researcher 3 was carried out to achieve the ability necessary to correctly implement such measurements.

Questionnaires were collected at day 5 after surgical exodontia. Trismus was assessed objectively by calculating the difference between the initial maximum interincisal opening, prior to anaesthetizing the patient, and the final oral opening 72 hours after surgery by means of a vernier caliper calibrated in millimeters.

A series of intraoperative and postoperative variables were also collected, including gender, patient’s age (in years). Degree and type of impaction of the third molar in accordance with the classification by Pell and Gregory (9), the angle regarding the second lower molar and the degree of complexity of the extraction according to Koerner index (10) were assessed by two of the researchers (Op1 and Op2, previously trained). They were calibrated obtaining a level of agreement of 86.41% (kappa = 0.63), 87.56% (kappa = 0.72) and 89.1% (kappa = 0.86) respectively between them (95% CI).

Other intraoperative variables collected were the quantity, in cubic centimeters, of local anesthetic administered, the total duration of surgery (from the moment of incision to the last stitch), the time in which the first sign of the anesthetic effect appeared after injection, the actual time for ostectomy and tooth sectioning simply counting the seconds during which the drill was working on the bone or tooth, as well as the total time to obtain the dislocation of the M3, alveolar curettage and suture.

The number of capsules or Tablets for the rescue analgesic treatment (metamizole magnesium in 575mg capsules) and the oral opening after 72 hours were among the variables included in the postoperative course, as well as the recording of other aforementioned facial distances.

The surgical extraction of the lower M3s was performed by following the usual technique, administering local anesthesia and performing inferior alveolar nerve block by means of direct technique, and injecting 4% articaine with 1:100,000 epinephrine (Artinibsa® by Laboratorios Inibsa Dental, Barcelona, Spain) in the buccal cavity through the same type of needle (Monoprotect® 30G/0.3x25mm by Laboratorios Inibsa Dental, Barcelona, Spain). If necessary during surgery, intrapulpal, intraligamentary or infiltrative anesthesia was injected as an anesthetic booster. The total quantity of anesthetic agent should not exceed three 1.8mL compules (5.4mL of anesthetic solution) otherwise the patient was withdrawn from the study.

A sterile field was prepared and, in order to obtain a partial Neumann mucoperiosteal flap, a distal incision of 2cm in the second lower molar was carried out (with number 3 scalpel handle and number 15 blade), as well as an intrasulcular incision around this tooth and a vertical incision with vestibular release which was retracted by means of Minnesota retractor. In addition, if necessary, the lingual flap was separated with Freer periosteal elevator.

To remove the M3 ostectomy was performed, as well as odontosection of crown and roots if necessary, to obtain the dislocation and extraction of the M3. Immediately thereafter, the operative area was curetted—both the alveolus and the space between the periosteum and the mandibular buccal cortical bone were washed with pressurized sterile saline solution. For the suture, 3/0 silk thread was used with C-16 atraumatic needle.

Each of the operative times, i.e. the time elapsed for the anesthesia to take effect, incision time and flap de-tachment, ostectomy, tooth sectioning and dislocation–avulsion and suture, were timed by operator 3.

-Statistical Analysis

Data were analyzed through the statistical program Stata (StataCorp. 2015. Stata Statistical Software: Release 14. College Station, StataCorp LP, Texas, USA). Means and percentiles were calculated to describe quantitative variables, and percentages to describe categorical variables. With the purpose of analyzing the differences between operators, the median test was used for paired data, since variables often had asymmetric distributions, and in some cases the Stuart–Maxwell test for marginal homogeneity was applied. Concerning the analyzed variables, the percentage of times (or interventions) the most experienced surgeon (operator 1) was better than the least experienced (operator 2) was calculated as well. Comparisons with *p* value <0.05 were considered statistically significant.

## Results

A total of 50 patients were included in the study, 32 men (64%) and 18 women (36%). Each surgeon operat-ed on one of the two lower third molars—a total of 100 surgical extractions were performed.

The age of patients ranged from 18 to 37 years, with a mean age of 23.44 (SD±5.19) years. Reason for the extraction: 32% (Op1) and 40% (Op2) were asymptomatic but with pathology derived from malposition (rhizolysis of adjacent molar, caries, pericoronitis, etc.). The least prevalent indications were dental crowding and recurrent episodes of pericoronitis by 8% for both operators. No significant differences were observed between operators in terms of extraction indications. In relation to the number of anesthetic compules, both operators administered a minimum of 1.5 anesthetic compules, although Op1 administered more anesthesia (2.28 vials) than Op2, being the average time until anesthesia began to take effect shorter for Op1 (3.12 minutes) compared to Op2 (4.6 minutes). However, there were no statistically significant differences (*p*=0.16).

The total average time for surgeries was 21.76 (SD±7.68) minutes. Table 1 shows how, in each of the parts the surgery consists of, the time used by Op2 was always slightly longer than by Op1. Tooth sectioning, which was not an inclusion criterion, was not necessary in 8 extractions for each operator. Without increasing the total time, the means were 1.14 minutes and 1.98 minutes for Op1 and Op2, respectively. In 84% of patients belonging to Op2 group, the time elapsed for ostectomy and/or tooth sectioning was significantly longer (*p*<0.00005). In terms of overall time, this was higher in all surgeries by Op2 (mean: 25.8 min) than in those by Op1 (mean: 17.8 min), being the difference between operators statistically significant (*p*=0.00005) (Table 1).

**Table 1:**
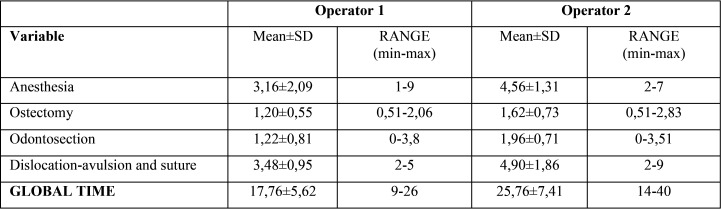
Time used by each operator in each part of the surgery (in minutes).

The career as an oral surgeon was taken into account to assess the degree of expertise of operators. In rela-ting this aspect to the time needed in each surgery—considering factors such as the degree of surgical difficulty, etc.—it is observed that in general Op1 always needed less time than Op2 (*p*=0.00005). The lower the level of expertise, the higher the time elapsed when working regardless of the degree of difficulty (*p*<0.05).

Nevertheless, if we analyze the overall time for surgery based on the degree of difficulty of extractions (ac-cording to Koerner index), such time was higher for Op2 than for Op1 in 92% of patients (*p*<0.00005). This percentage was 100%, 75% and 100% when the degree of difficulty was 1, 2 and 3 respectively, being the differences between operators statistically significant (*p*<0.0005). In relation to the degree of difficulty, the Kappa index between both operators was 91% (*p*<0.00005).

The minimum value for pain indicated in VAS was 0 and the maximum was 10, i.e. there were patients who reported pain rated as unbearable, although only four patients treated by Op1 reported this maximum pain at hour 4 and 5 in the postoperative period.

**Figure 2:**
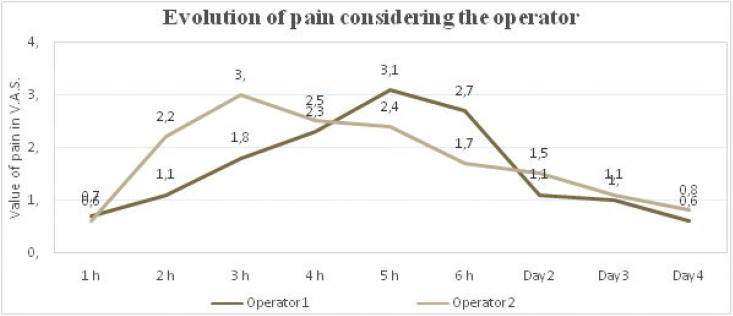
Evolution of pain after surgical removal considering the experience of the operator.

The mean value for pain indicated in VAS was similar during the first hour post-extraction (0.6 and 0.7 for Op1 and Op2, respectively), but with statistically significant differences (*p*<0.05) at hour 1, 2 and 3, being pain greater during the first hour in nearly 88% of surgeries by Op1, whereas during the second and third hour post-surgery pain was greater in patients from Op2 group (Fig. 2). On the first day post-surgery there were statistically significant differences (*p*<0.001) regarding postoperative pain between the two operators. On the second day both in the morning and at night, pain was greater in participants treated by Op2 than in those treated by Op1, whereas on the last day, day 4, pain was slightly greater in those patients treated by Op1, with no statistically significant differences on any of those days (Table 2).

**Table 2:**
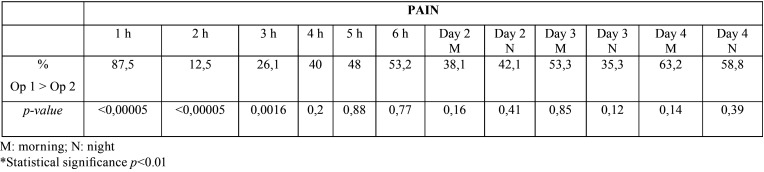
Percentage of times pain was greater with operator 1 that with operator 2. Statistical differences between operator 1 and 2.

With regard to the consumption of analgesics, 69.2% of patients took more analgesics with Op1 than with Op2, with a mean of 1.8 and 1, respectively (*p*=0.0755). With respect to the total number of surgeries, the dif-ference in inflammation (facial measurements) was 0.84 (SD±19.94) mm, whereas the mean for trismus was 9.78 (SD±7.69) mm. When analyzing the increase in facial measurements at 72 hours, it is observed that in the case of Op1 the postoperative facial edema was almost imperceptible, while in the case of Op2 differences were detected between preoperative and postoperative inflammation, with statistical significance compared to the most experienced operator (*p*=0.040).

The degree of facial inflammation expressed by means of the difference between the preoperative and postoperative measurements was 0.7 and 4.32mm for Op1 and Op2, respectively; i.e. it was not statistically significant (*p*=0.8877) (Table 3). Fifty-two per cent (52%) of times, patients treated by Op2 had greater facial inflammation. The difference in the degree of oral opening or trismus was greater in surgeries performed by Op1 (mean: 10.7 mm) compared to those by Op2 (mean: 8.9mm), (*p*=0.040).

**Table 3:**
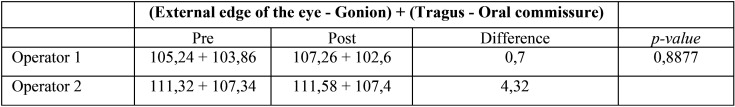
Differences between pre and post-operative facial measures for each operator (millimeters).

## Discussion

During the postoperative period in surgical extraction of impacted M3s, pain, swelling and trismus occur. They depend on many variables analyzed in our work, including the operator’s degree of expertise.

Although in this study the impact of age regarding intraoperative complications was not analyzed, we must indicate that the sample comprised young patients between 18 and 37 years of age. Several authors state that the removal of third molars is considerably less complicated when performed in young adults, with increased complications in patients over 50 years of age (8,11,12). However, other authors did not find a significant relationship between age and operative time, and the incidence of postoperative complications (13).

The influence of patient’s gender was not related to the occurrence of complications either. Many studies have shown that women reported greater pain in VAS after the extraction of the M3 (11), although these results considerably differ from those obtained by Alvira-González *et al.* (12) and the study by Pell and Gregory (9), without observing differences between genders.

As regards the reasons for the extraction, we concluded that for both operators the major indication was asymptomatic M3s along with pathology (caries, a lesion presumptively diagnosed as cyst, etc.), 32% of cases for Op1 and 40% for Op2, whereas in very few cases the extraction was due to recurrent episodes of pericoro-nitis. These data coincide on those of Fuster-Torres *et al.* (6) or of Marques *et al.* (19), who recommend preven-tative extraction due to the high prevalence of caries (20.6–30.2%) occurring in the distal aspect of the second molar. For their part, Alves-Pereira *et al.* (1) highlight that the main reason for extraction is the prevention of pericoronitis, closely associated with M3 impaction.

In the present study, in which 4% articaine with 1:100,000 epinephrine was used as an anesthetic solution, Op1 administered a mean of 2.28 compules while Op2 utilized 2.18 compules (*p*>0.05). These data are similar to those obtained in the study by Benediktsdóttir *et al.* (20).

The estimation of the degree of difficulty for the extraction of M3s is necessary to reduce the surgery time. In our work, the difficulty index was assessed by both operators, having previously carried out a concordance test. Authors such as Ferrús-Torres *et al.* (21) confirm that clinical expertise in foreseeing surgical difficulty is a very important factor for the postoperative period, and mistakes when estimating such degree decrease as surgi-cal expertise increases. Poor surgical planning could lead to intraoperative events and, therefore, increase the time for surgery. Other authors confirm that surgeons with lower expertise level make many more mistakes when preoperatively assessing the extraction difficulty (8). However, Komerik *et al.* (22) found no relation with exper-tise and the prediction in the degree of surgical difficulty.

As regards the surgery time, in our study the most experienced operator (Op1) performed the extractions in less time than Op2 in 100% of surgeries with *p*<0.005; results shared with Susarla and Dodson (15), who also concluded that the surgical experience and the duration of surgery had a negative correlation, i.e. the higher the expertise level, the shorter the operative time. Other studies prove that if the M3 extraction is carried out by professionals with a lot of experience, the operative time is reduced significantly (13). Some authors even state that, per each year of operator’s experience, the operative time is reduced at a 0.20 minutes/year rate (15).

Regarding the position of the M3, the greater the bone depth, the greater the difficulty in removing it, the duration of surgery, inflammation and trismus (23). Nevertheless, the level of patient’s postoperative pain or the need for analgesics according to the type of impaction, are very difficult to foresee. Severe impaction forces the surgeon to perform greater ostectomies and implement more complicated extraction maneuvers, although au-thors like Fisher *et al.* (24) found no increased pain when comparing patients with partially erupted and unerupted third molars.

Studies by Berge and Boe (23) showed that swelling increases as the operative time increases, being especially important when surgery exceeds 15 minutes time.

Measurements on the patient’s face were already described by Amin and Laskin (18) in 1983 to assess and objectify inflammation. In our study inflammation was only measured before surgery and at 72 hours post-surgery, since our objective was to see who of the two operators caused more inflammation. The mean oral opening ranges between 32mm and 77mm in adults (25). However, in our study the most experienced operator obtained a mean reduction of 10.7mm in postoperative opening, whereas the least experienced operator obtained a mean reduction of 8.9mm in postoperative opening, i.e. trismus was always higher in patients treated by Op1, specifically 66% of the time, establishing a statistically significant difference (*p*<0.05). However, for Jerjes *et al.* (26) the incidence of trismus was higher in the group of less experienced surgeons. On the other hand, Tenglikar *et al.* (27) obtained a statistically significant relationship between inflammation and trismus regarding the opera-tive time.

Many factors, such as gender, age, patient’s anxiety, surgical difficulty (22), and even the operator’s degree of expertise, influence pain. A study that assessed the intensity of postoperative pain after M3 extraction showed that the patient’s age, operative time, the surgeon’s degree of expertise, the occurrence or absence of pericoronitis and the degree of impaction did not influence the severity of postoperative pain, being the patient’s gender the only factor which used to be statistically significant (24).

The operative time is considered to be determined by the position of the molar and the surgeon’s expertise. These parameters would determine, therefore, the difficulty of the procedure, since they would intensify edema, inflammation and the duration of pain (22). On the other hand, Tenglikar *et al.* (27) obtained a statistically significant relationship between pain and the duration of surgery. However, according to Van Gool *et al.* (28), the severity of pain after removing the lower M3s does not seem to be related to the type of incision, the amount of ostectomy or the need for tooth sectioning.

In addition, there are studies that relate the professional’s little experience to increased pain, and consequently to increased consumption of analgesics after surgery (14), whereas studies like those by Rakhshan *et al.* (7) do not find any relation between the operator’s expertise and postoperative pain. Furthermore, pain and analgesic intake increased when surgical trauma increased (29). In our study patients operated on by the most experienced surgeon received in 69.2% of cases more rescue analgesic treatment than those treated by the least experienced operator.

After analyzing the results, the conclusion drawn is that there are differences between the two operators regarding the course of postoperative period after surgical removal of impacted lower M3s. With respect to the time needed by each of the operators, the most experienced operator always completed the surgery in less total time than the least experienced operator, and concerning the inflammation occurring in the postoperative period no differences between both of them were detected in terms of facial measurements. Trismus, however, was greater in those patients treated by the operator with more experience. As regards pain, significant differences were found between the patients of the two surgeons during the first three hours of postoperative period, being this more intense in those operated on by Op1 (more experienced).

Concerning the extraction of an impacted lower M3 with certain surgical difficulty, the postoperative course of patients operated on by a surgeon with more experience does not foreseeably differ very much in the results compared to those of a less experienced operator, although the former requires less time to complete surgery.

The major problem found in the reviewed literature is that very few studies take into account the operator’s degree of expertise and those considering such degree do not specify neither the training each operator had in this field, nor the years of professional experience in Oral Surgery. No designs of detailed studies are made either because studies are not conducted by following the split–mouth design.

We conclude that, although Op2 needs more time when performing any surgery due to less professional experience compared to that of Op1, even with equal difficulty degrees, his/her postoperative period does not entail more inflammation or pain in patients, so there are no relevant differences between surgeons with more or less experience. The skills and training received are the factors influencing to a greater extent the operative process.

The main limitation of our study was the sample size because we needed the same patient to undergo the extraction of the two lower third molars, with similar complexity (according to Koerner’s index), and on two different days. For this reason, in order to have preliminary data, we decided to conduct a pilot study. In prospective research we would like to increase the sample size and compare two large groups of oral surgeons, each one of them with different experiences in oral surgery, and even to compare other oral surgery services from other faculties with ours.
